# Reduced Susceptibility to Carbapenems in a Klebsiella pneumoniae Clinical Isolate Producing SCO-1 and CTX-M-15 β-Lactamases Together with OmpK35 and OmpK36 Porin Deficiency

**DOI:** 10.1128/AAC.00556-20

**Published:** 2020-07-22

**Authors:** Carolina Venditti, Ornella Butera, Anna Proia, Luigi Rigacci, Bruno Mariani, Gabriella Parisi, Francesco Messina, Alessandro Capone, Carla Nisii, Antonino Di Caro

**Affiliations:** a Laboratory of Microbiology, National Institute for Infectious Diseases L. Spallanzani, IRCCS, Rome, Italy; b Haematology and Haematopoietic Stem Cells Transplant Unit, San Camillo-Forlanini Hospital, Rome, Italy; c Laboratory of Microbiology and Virology, San Camillo Forlanini Hospital, Rome, Italy; d Severe and Immunocompromised-associated Infections Unit, National Institute for Infectious Diseases L. Spallanzani, IRCCS, Rome, Italy

**Keywords:** ESBLs, *Klebsiella pneumoniae*, OmpK35, OmpK36, SCO-1, carbapenem resistance, porin deficiency

## LETTER

Extended-spectrum beta-lactamases (ESBLs) and carbapenemases are among the most important causes of resistance in *Enterobacterales*, often leading to health care-associated infections that result in high morbidity and mortality (1–3). Resistance to carbapenems in particular may be the result of a number of mechanisms, including the production of serino- and/or metallo-beta-lactamases (mainly KPC, VIM, and NDM), but can also result from the production of ESBLs and/or AmpC β-lactamases in association with alterations in the expression of outer membrane porins (OMPs) (4–6).

Here, we describe a case of carbapenem resistance in a Klebsiella pneumoniae strain isolated from a 58-year-old male with a diagnosis of acute myeloid leukemia treated at the San Camillo-Forlanini Hospital in Rome, Italy. During his hospitalization, the patient developed a bloodstream infection caused by a carbapenem-resistant (CR) K. pneumoniae strain; immediately after the diagnosis, a combination therapy with ceftazidime-avibactam and amikacin was started, with prompt clinical improvement.

The strain was identified as K. pneumoniae by matrix-assisted laser desorption ionization–time of flight mass spectrometry (MALDI-TOF MS; bioMérieux, Marcy l’Étoile, France); antimicrobial susceptibility was obtained by Phoenix system (Becton, Dickinson Diagnostics, CA). MICs were interpreted according to the European Committee on Antimicrobial Susceptibility Testing (EUCAST) recommendations (7). MICs for tigecycline and for cefotaxime-clavulanic acid, imipenem-relebactam, and meropenem-vaborbactam were confirmed by MIC gradient strip (MIC test strip; Liofilchem, Roseto degli Abruzzi, Italy); MICs for colistin and ceftazidime-avibactam were confirmed by broth microdilution (Sensititre; Thermo Fisher Scientific, USA). The antimicrobial susceptibility profile showed that this carbapenem-resistant isolate was susceptible to beta-lactam–beta-lactamase inhibitor combinations (which is consistent with the successful treatment of the patient with ceftazidime-avibactam), as well as tigecycline, colistin, and amikacin (Table 1). Confirmatory phenotypic inhibition tests for detection of serino- and/or metallo-beta-lactamase production were performed using phenylboronic acid (PBA) and EDTA (Liofilchem), respectively. Despite a positive result for serino-beta-lactamase production, the rapid immunochromatographic assay (NG-test Carba; NG Biotech), carried out to confirm the presence of the most common carbapenemases (KPC, NDM, VIM, IMP, and OXA-48-like enzymes), provided a negative reaction, demonstrating the absence of KPC and OXA-48-like enzymes.

**TABLE 1:** Phenotypic and molecular analysis of the K. pneumoniae clinical isolate described here^*a*^

Drug	MIC (mg/liter) and susceptibility
AMK	<4, S
CAZ	>8, R
CIP	>1, R
ETP	>32, R
MEM	12, R
IPM	8, R
GEN	>4, R
TZP	>64/4, R
SXT	>4/76, R
CST	0.5, S
TGC	1, S
CZA	2/4, S
CTL	1/4, S
I-R	0.75/4, S
MVB	1/4, S

a AMK, amikacin; CAZ, ceftazidime; CIP, ciprofloxacin; ETP, ertapenem; MEM, meropenem; IPM, imipenem; GEN, gentamicin; TZP, piperacillin-tazobactam; SXT, trimethoprim-sulfamethoxazole; CST, colistin; TGC, tigecycline; CZA, ceftazidime-avibactam; CTL, cefotaxime-clavulanic acid; I-R, imipenem-relebactam; MVB, meropenem-vaborbactam; S, susceptible; R, resistant.

To investigate further, whole-genome sequencing (WGS) was performed using the Ion Torrent GSS5 platform (Life Technologies, Carlsbad, CA) by constructing single-end libraries with average length of 200 bp according to the manufacturer’s instructions. Antimicrobial resistance genes and plasmid replicons were extracted from the WGS data identified by the *in silico* analysis using the ResFinder v3.0 web server (http://www.genomicepidemiology.org) (8); clonality was analyzed by the traditional multilocus sequence typing (MLST) with seven housekeeping genes (http://bigsdb.pasteur.fr/klebsiella/klebsiella.html). Bioinformatics analysis showed that the CR K. pneumoniae strain belonged to sequence type 15 (ST15) and harbored, in addition to *bla*_OXA-1_, *bla*_SHV-28_, *bla*_TEM-1_, and *bla*_CTX-M-15_ beta-lactamase genes, also *bla*_SCO-1_. The following resistance-encoding genes were also present: *aac*(3)-IIa and *aadA*1 for aminoglycosides; *fosA* for fosfomycin; *aac*(*6*′)-Ib-cr, *oqxA*, *oqxB*, and *qnrB2* for quinolones; *catB3* for phenicol; *sul1* for sulfonamide; the *tet*(D) gene, encoding efflux pumps responsible for tetracycline resistance, and *dfrA15* for resistance to trimethoprim. The analysis performed by PlasmidFinder showed the presence of the incompatibility (Inc) group IncHI1B replicon pNDM-MAR (GenBank accession no. JN420336), with 100% identity. This plasmid had been previously isolated from a clinical K. pneumoniae ST15 isolate harboring *bla*_NDM-1_ and *bla*_CTX-M-15_ (9). Although we did not perform an in-depth plasmid sequence analysis, the fact that these repHI1B plasmid sequences (GenBank accession no. CP037442.1 and CP042866), harboring the *bla*_SCO-1_ and *bla*_TEM-1_ genes, have been found in K. pneumoniae and Escherichia coli supports the hypothesis that also in our K. pneumoniae the identified repHI1B plasmid could harbor both resistance genes.

The major OMP genes, OmpK35 and OmpK36, were analyzed by PCR to investigate membrane permeability deficiency; the amplification products of the genes were sequenced and analyzed using the NCBI BLAST program (https://www.ncbi.nlm.nih.gov/BLAST). The results showed a clear genetic disruption for both of the *ompK35* and *ompK36* genes; further analysis revealed a nonfunctional *ompK35* gene and loss of OmpK36.

The combination of a membrane permeability deficiency (caused by a nonfunctional *ompK35* gene and the loss of *ompK36*) with beta-lactamase enzyme carriage has already been shown to reduce susceptibility to carbapenems in *Enterobacterales*, especially in K. pneumoniae (5, 10). The product of the *bla*_SCO-1_ gene, in particular, is a plasmid-mediated class A carbenicillinase of unknown origin (11–13) able to hydrolyze not only penicillins but also, to a lesser degree, cephalosporins and carbapenems. Since its discovery in 2007 (12), the *bla*_SCO-1_ gene (GenBank accession no. EF063111) has been identified (out of the 31,614 whole-genome sequences present in the BLAST database) in Acinetobacter baumannii, Escherichia coli, Serratia marcescens, Klebsiella aerogenes, Salmonella enterica, and only four K. pneumoniae isolates (11–17). It is likely that the real prevalence of the enzyme in Gram-negative bacteria is largely underestimated due to the lack of epidemiological studies and because it is not part of any routine screening for resistance genes. While the increased carbapenem MICs observed in our study may well be the result of “classical” ESBL enzymes in association with porin deficiency, as already described (4–6, 10), the finding of the *bla*_SCO-1_ gene highlights the risk of transmission also of uncommon plasmid-borne resistance genes. Although the reporting of such events is still low, their occurrence could be more frequent than thought and deserves careful monitoring. The potential spread of clinical strains expressing unusual ESBLs associated with OMP deficiency could represent a problem for carbapenem-resistant *Enterobacteriaceae* (CRE) surveillance schemes. Many multiplex-based molecular screening assays for the most common resistance genes are becoming commercially available, and in an era of ever-increasing workloads and attention to budget, laboratorians could be tempted to screen rectal swabs by molecular methods only, performing cultures only on positive samples. If such a procedure were employed, a strain like the one described here would be missed.

In conclusion, we have described the occurrence of a CR K. pneumoniae strain expressing an unusual ESBL enzyme, leading to bacteremia in an immunosuppressed patient. Routine diagnostic tests were unable to explain this carbapenem resistance, apart from a positive result for serino-beta-lactamase production, while a deeper molecular characterization using WGS allowed a more comprehensive picture of antimicrobial resistance. Our results are a reminder, if needed, of the importance of relying on molecular or screening tests always in combination with MIC-based susceptibility testing and phenotypic tests, at least for critical samples screened for hospital surveillance.

## 

### Data availability.

Generated raw reads were submitted to the Sequence Read Archive (SRA) under BioProject accession number PRJNA612823 and SRA number SRX7915095.

## References

[1] Tacconelli E, Carrara E, Savoldi A, Harbarth S, Mendelson M, Monnet DL, Pulcini C, Kahlmeter G, Kluytmans J, Carmeli Y, Ouellette M, Outterson K, Patel J, Cavaleri M, Cox EM, Houchens CR, Grayson ML, Hansen P, Singh N, Theuretzbacher U, Magrini N, WHO Pathogens Priority List Working Group 2018 Discovery, research, and development of new antibiotics: the WHO priority list of antibiotic-resistant bacteria and tuberculosis. Lancet Infect Dis 18:318–327. doi:10.1016/S1473-3099(17)30753-3. 29276051

[2] Giani T, Antonelli A, Caltagirone M, Mauri C, Nicchi J, Arena F, Nucleo E, Bracco S, Pantosti A, AMCLI-CoSA survey participants, Luzzaro F, Pagani L, Rossolini GM 2017 Evolving beta-lactamase epidemiology in Enterobacteriaceae from Italian nationwide surveillance, October 2013: KPC-carbapenemase spreading among outpatients. Euro Surveill 22:30583. doi:10.2807/1560-7917.ES.2017.22.31.30583. 28797330 PMC5553057

[3] Paterson DL, Bonomo RA 2005 Extended-spectrum beta-lactamases: a clinical update. Clin Microbiol Rev 18:657–686. doi:10.1128/CMR.18.4.657-686.2005. 16223952 PMC1265908

[4] Martínez-Martínez L 2008 Extended-spectrum beta-lactamases and the permeability barrier. Clin Microbiol Infect 14:82–89. doi:10.1111/j.1469-0691.2007.01860.x. 18154531

[5] Hamzaoui Z, Ocampo-Sosa A, Fernandez Martinez M, Landolsi S, Ferjani S, Maamar E, Saidani M, Slim A, Martinez-Martinez L, Boutiba-Ben Boubaker I 2018 Role of association of OmpK35 and OmpK36 alteration and bla(ESBL) and/or bla(AmpC) genes in conferring carbapenem resistance among non-carbapenemase-producing *Klebsiella pneumoniae*. Int J Antimicrob Agents 52:898–905. doi:10.1016/j.ijantimicag.2018.03.020. 29621592

[6] Wise MG, Horvath E, Young K, Sahm DF, Kazmierczak KM 2018 Global survey of *Klebsiella pneumoniae* major porins from ertapenem non-susceptible isolates lacking carbapenemases. J Med Microbiol 67:289–295. doi:10.1099/jmm.0.000691. 29458684

[7] European Committee on Antimicrobial Susceptibility Testing (EUCAST). 2018 Breakpoint tables for interpretation of MICs and zone diameters, version 8.1, 2018. http://www.eucast.org.

[8] Carattoli A, Zankari E, García-Fernández A, Voldby Larsen M, Lund O, Villa L, Møller Aarestrup F, Hasman H 2014 In silico detection and typing of plasmids using PlasmidFinder and plasmid multilocus sequence typing. Antimicrob Agents Chemother 58:3895–3903. doi:10.1128/AAC.02412-14. 24777092 PMC4068535

[9] Villa L, Poirel L, Nordmann P, Carta C, Carattoli A 2012 Complete sequencing of an IncH plasmid carrying the blaNDM-1, blaCTX-M-15 and qnrB1 genes. J Antimicrob Chemother 67:1645–1650. doi:10.1093/jac/dks114. 22511638

[10] García-Fernández A, Miriagou V, Papagiannitsis CC, Giordano A, Venditti M, Mancini C, Carattoli A 2010 An ertapenem-resistant extended-spectrum-beta-lactamase-producing *Klebsiella pneumoniae* clone carries a novel OmpK36 porin variant. Antimicrob Agents Chemother 54:4178–4184. doi:10.1128/AAC.01301-09. 20660683 PMC2944588

[11] Matagne A, Lamotte-Brasseur J, Frère J-M 1998 Catalytic properties of class A beta-lactamases: efficiency and diversity. Biochemistry J 330:581–598. doi:10.1042/bj3300581. PMC1219177 9480862

[12] Poirel L, Corvec S, Rapoport M, Mugnier P, Petroni A, Pasteran F, Faccone D, Galas M, Drugeon H, Cattoir V, Nordmann P 2007 Identification of the novel narrow-spectrum beta-lactamase SCO-1 in *Acinetobacter* spp. from Argentina. Antimicrob Agents Chemother 51:2179–2184. doi:10.1128/AAC.01600-06. 17420213 PMC1891420

[13] Papagiannitsis CC, Tzouvelekis LS, Kotsakis SD, Tzelepi E, Miriagou V 2011 Sequence of pR3521, an IncB plasmid from *Escherichia coli* encoding ACC-4, SCO-1, and TEM-1 beta-lactamases. Antimicrob Agents Chemother 55:376–381. doi:10.1128/AAC.00875-10. 20956594 PMC3019680

[14] Jin W, Wachino J, Kimura K, Yamada K, Arakawa Y 2015 New plasmid-mediated aminoglycoside 6′-N-acetyltransferase, AAC(6′)-Ian, and ESBL, TLA-3, from a Serratia marcescens clinical isolate. J Antimicrob Chemother 70:1331–1337. doi:10.1093/jac/dku537. 25576529

[15] Ktari S, Arlet G, Verdet C, Jaoua S, Kachrid A, Ben Redjeb S, Mahjoubi-Rhimi F, Hammami A 2009 Molecular epidemiology and genetic environment of acquired *bla* ACC-1 in *Salmonella enterica* serotype Livingstone causing a large nosocomial outbreak in Tunisia. Microb Drug Resist 15:279–286. doi:10.1089/mdr.2009.0035. 19857134

[16] Nadjar D, Rouveau M, Verdet C, Donay L, Herrmann J, Lagrange PH, Philippon A, Arlet G 2000 Outbreak of *Klebsiella pneumoniae* producing transferable AmpC-type beta-lactamase (ACC-1) originating from Hafnia alvei. FEMS Microbiol Lett 187:35–40. doi:10.1111/j.1574-6968.2000.tb09133.x. 10828397

[17] Doloy A, Verdet C, Gautier V, Decré D, Ronco E, Hammami A, Philippon A, Arlet G 2006 Genetic environment of acquired *bla*(ACC-1) beta-lactamase gene in Enterobacteriaceae isolates. Antimicrob Agents Chemother 50:4177–4181. doi:10.1128/AAC.00619-06. 16982793 PMC1693989

